# Fertility concerns and treatment decision‐making among national sample of young women with breast cancer

**DOI:** 10.1002/cam4.6838

**Published:** 2023-12-22

**Authors:** Elisabeth de Kermadec, Yue Zheng, Shoshana Rosenberg, Kathryn J. Ruddy, Jennifer A. Ligibel, Karen M. Emmons, Ann H. Partridge

**Affiliations:** ^1^ Medical Oncology Dana‐Farber Cancer Institute Boston Massachusetts USA; ^2^ Breast Oncology Program Dana‐Farber Brigham Cancer Center Boston Massachusetts USA; ^3^ Data Science Dana‐Farber Cancer Institute Boston Massachusetts USA; ^4^ Harvard Medical School Boston Massachusetts USA; ^5^ Mayo Clinic Rochester Minnesota USA; ^6^ Harvard T. H. Chan School of Public Health Boston Massachusetts USA; ^7^ Present address: Weill Cornell Medicine New York New York USA; ^8^ Present address: Sanofi Cambridge Massachusetts USA

**Keywords:** breast cancer, decision‐making, fertility, fertility concerns and preservation strategies, young women

## Abstract

**Background:**

Diagnosis of breast cancer in young women has been shown to affect their decision‐making with regard to fertility and family planning. Limited data are available from populations across the U.S. regarding this issue; thus, we sought to describe fertility concerns and efforts to preserve fertility in a national clinical trial population of young breast cancer patients.

**Methods:**

The young and strong study was a cluster‐randomized controlled trial testing an intervention program for young women with breast cancer. Patients were surveyed within 3 months after diagnosis and at 3, 6, and 12 months after. Surveys asked about sociodemographics, psychosocial domains, fertility concerns, and fertility preservation strategies. Univariable and multivariable models were used to investigate sociodemographic, clinical, and psychosocial predictors of fertility concerns.

**Results:**

Of 467 women from 54 clinical sites across the U.S. (14 academic, 40 community), 419 were evaluable regarding fertility concerns. Median age was 40 years (range 22–45), 11% were Black, 6% Hispanic, and 75% had children. Tumor stage was I (35%), II (51%), or III (14%); 82% received chemotherapy. At time of the treatment decision, 133 (32%) participants had fertility concerns, among whom 47% indicated this affected their treatment decisions. Sixty percent of participants reported having discussed fertility with their physician. Twenty percent of those with fertility concerns used fertility preservation strategies. History of difficulty becoming pregnant and younger age were associated with higher odds of fertility concerns in multivariable modeling.

**Conclusion:**

Many young women with newly diagnosed breast cancer are concerned about fertility in a way that impacts their treatment decisions. Concerns were discussed, but few used fertility preservation strategies. These findings have implications for counseling young patients.

## 1 INTRODUCTION

Approximately 10% of new breast cancer diagnoses in the U.S. affect women age 45 years and younger.^1^ Tumors in this population are more likely to be aggressive, and intensive therapies including multiagent chemotherapy are frequently prescribed. Fertility can be affected by the direct gonadotoxic effects of chemotherapy or by the requisite delay of conception until the completion of adjuvant therapy, which is generally 5–10 years with standard endocrine therapy.^2^

As postponement of pregnancy to the 30s and 40s is becoming more common in the general population,^3^ it is more likely that a young woman who is diagnosed with breast cancer may not have started or completed her family and may harbor concerns about treatment‐related infertility. Attention to this issue for all adolescent and young adult cancer patients is now recommended by international guidelines,^4^, ^5^, ^6^ and recent studies suggest that there is still sub‐optimal attention to fertility.^7^, ^8^, ^9^, ^10^, ^11^, ^12^ Even when fertility is addressed, a patient's options and access to, as well as effectiveness of fertility preservation strategies may be limited.^13^ Fertility concerns can also affect treatment decisions including choice of or forgoing chemotherapy, non‐initiation, or non‐persistence with adjuvant hormonal therapy, in particular.^14^, ^15^, ^16^

Some prior studies have examined fertility concerns in young breast cancer patients. In one European study led by Ruggieri et al., among 297 patients surveyed when they were making treatment decisions, 34% reported no concerns about becoming infertile.^15^ While Ruddy et al.^14^ described the association of race with fertility concerns among 620 participants, this population was geographically restricted, and only 3% of participants were Black. We sought to better understand the prevalence of fertility concerns, factors associated with these concerns, and how fertility concerns affect treatment decisions and fertility preservation strategies at the time of decision‐making in a more diverse national population of young women with breast cancer from across the U.S.

## 2 MATERIALS AND METHODS

### 2.1 Study design

The present study is a secondary data analysis from a prospective clinical trial, the Young and Strong study (NCT01647607^17^). This cluster‐randomized trial tested the effect of an educational and supportive care intervention for young women with breast cancer (diagnosed age ≤45 years) and their oncologists designed to improve attention to fertility. The study was conducted across the U.S. at 14 academic and 40 community practices which were randomized to either the young women's intervention (YWI) or to an attention control physical activity intervention (PAI). Participants provided written informed consent prior to enrollment. Institutional review board approval for the study was obtained from the Dana‐Farber/Harvard Cancer Center and other participating institutions.

Between July 2012 and December 2013, 467 English‐speaking women aged 18 to 45 years with newly diagnosed breast cancer were enrolled within 3 months of diagnosis. The primary results of the young and strong study are reported in detail elsewhere.^12^ In brief, the study did not demonstrate differences in attention to fertility, as assessed by medical record review, between the two intervention groups, with similar rates in both arms (55% for the YWI group vs. 58% for the PAI group, *p* = 0.88).

Thus, for the current analysis, in which we sought to describe the actual fertility concerns of the participants and related issues, the two arms were combined, and we used data from surveys completed by study participants at baseline (within 3 months of diagnosis, mean of 46 days), 3, 6, and 12 months after enrollment.

### 2.2 Outcome measures

The primary outcome of this analysis was concerns about fertility when making breast cancer treatment decisions as assessed by one question on the 3‐month survey. Women were asked “When you were making breast cancer decisions about your treatment, how concerned were you about the possibility of becoming infertile?” and categorized as concerned if they responded “a little,” “somewhat,” or “very,” or not concerned if they responded “not at all” to the question.

Secondary outcomes, using items from a refined version of the fertility issues survey (FIS),^18^ included: (1) use of fertility preservation prior to therapy (“no” or “unsure” vs. “yes” on the 3‐month survey in response to “Prior to cancer treatment, did you take any special steps to lessen the chance that you would become infertile with cancer treatment?”) and (2) impact of fertility concerns on treatment decisions (“a little”, “somewhat,” or “very” vs. “not at all” on the 3‐, 6‐, or 12‐month survey in response to “How much did your concern about becoming infertile after your cancer treatment affect your treatment decisions?”).

Covariates from the baseline survey included sociodemographics (age, ethnicity, education, marital status, employment status, and income) and assessed items from the Sociocultural Module of the USDHHS's Breast cancer Core Questionnaire.^19^, ^20^ Stress was measured using the Perceived Stress Scale (PSS) with categories of low (<14), moderate (14–26), or high (≥27) stress.^21^ Anxiety was measured from the Hospital Anxiety and Depression Scale (HADS) anxiety subscale dichotomized as not anxious (normal [score <8] or borderline [scores 8–10]) vs. anxious (score ≥ 11).^22^, ^23^ Depression was measured from the HADS depression subscale and dichotomized as not depressed (normal [score <8] or borderline [score 8–10]) vs. depressed (score ≥ 11).^22^, ^23^

Tumor stage, estrogen and progesterone receptor (ER/PR) expression, human epidermal growth factor receptor 2 (HER2) overexpression, and treatment were ascertained by medical record review.

### 2.3 Statistical analysis

Demographic and clinical characteristics were analyzed using descriptive statistics. Stacked bar charts were plotted, and McNemar's test was used to show the change in fertility concerns over time. Univariate logistic regression was used to assess the association between fertility concerns and sociodemographics, tumor and treatment characteristics, psychosocial measures, and fertility‐related variables. Parameter estimates are reported as odds ratios (ORs) with 95% confidence intervals (CI) derived from robust standard errors. Variables where the *p*‐value was ≤0.20 in univariate analyses were evaluated in a multivariate logistic regression model using backward stepwise selection, and variables achieving significance at *p* ≤ 0.05 were included in the final model. Additional subgroup analysis was conducted among only those participants who were concerned about fertility assessed the association between patient/disease characteristics and treatment decisions affected by fertility concerns, as well as use of fertility preservation. Statistical analyses were performed in SAS 9.4 (SAS Institute Inc. Cary, NC).

## 3 RESULTS

**FIGURE 1:**
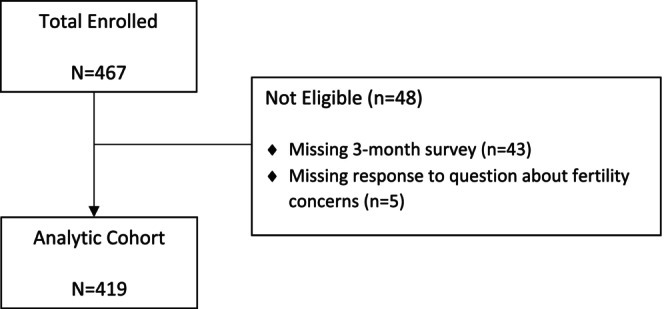
Study consort.

Of the 467 women enrolled, 48 (10%) did not respond to the 3‐month survey or to the questions regarding fertility at the time of treatment decision‐making and thus were excluded (Figure 1). Table 1 presents the patient and disease characteristics in the total analytic cohort (*n* = 419) and among the subset who indicated any fertility concerns (*n* = 133, including those reporting they were “a little” concerned [*N* = 46, 11%], “somewhat” concerned [*N* = 28, 6.7%], and “very” concerned [*N* = 59, 14.1%]). The median age of participants at enrollment was 40 years (range, 22–45). The majority of women were white (79%), 11% were Black, and 6% Hispanic. Most women were married or living with a partner (80%), had children (75%), and were college educated (88%). Chemotherapy was administered to the majority (82%). Thirty‐five percent of tumors were stage I, 51% stage II, and 14% stage III. Approximately one‐third of women reported high levels of anxiety, and two‐thirds reported moderate or high levels of stress.

**TABLE 1:** Patient demographic and clinical characteristics.

Characteristic	No. of patients (%)	
	Total cohort (*N* = 419)	Concerned cohort (*N* = 133)
Patient characteristics		
Age: median [range], years	40 [22–45]	35 [22–45]
Age (years)		
≤30	32 (7.6%)	29 (21.8%)
31–35	66 (15.8%)	43 (32.3%)
36–45	321 (76.6%)	61 (45.9%)
Race		
White	330 (78.8%)	102 (76.7%)
Black	45 (10.7%)	10 (7.5%)
Asian	18 (4.3%)	7 (5.3%)
Multiracial or other	24 (5.7%)	14 (10.5%)
Missing	2 (0.5%)	0 (0%)
Hispanic	27 (6.4%)	10 (7.5%)
College educated	367 (87.6%)	125 (94.0%)
Region of enrollment		
Northeast	103 (24.6%)	35 (26.3%)
Southeast	98 (23.4%)	23 (17.3%)
Midwest	150 (35.8%)	48 (36.1%)
Southwest	17 (4.1%)	5 (3.8%)
West	51 (12.2%)	22 (16.5%)
Site type		
Academic	165 (39.4%)	61 (45.9%)
Community	254 (60.6%)	72 (54.1%)
Employed	250 (59.7%)	83 (62.4%)
Earned income		
Less than $25,000	45 (10.7%)	11 (8.3%)
$25,000–$49,999	63 (15.0%)	29 (21.8%)
$50,000–$99,999	129 (30.8%)	50 (37.6%)
Above $100,000	151 (36.0%)	33 (24.8%)
Unknown or missing	31 (7.4%)	10 (7.5%)
Married or living as married	334 (79.7%)	101 (75.9%)
First‐degree relative with breast or ovarian cancer	69 (16.5%)	23 (17.3%)
Psychosocial measures		
PSS		
Low (<14)	156 (37.2%)	49 (36.8%)
Moderate (14–26)	232 (55.4%)	71 (53.4%)
High (≥27)	30 (7.2%)	13 (9.8%)
Missing	1 (0.2%)	0 (0%)
Anxiety (anxiety score ≥11)	134 (32.0%)	46 (34.6%)
Depression (depression score ≥11)	32 (7.6%)	10 (7.5%)
Tumor and treatment characteristics		
Stage of cancer		
I	145 (34.6%)	43 (32.3%)
II	213 (50.8%)	73 (54.9%)
III	58 (13.8%)	16 (12.0%)
IV	2 (0.5%)	1 (0.8%)
Missing	1 (0.2%)	0 (0%)
ER/PR positive	313 (74.7%)	98 (73.7%)
Her2 positive	101 (24.1%)	30 (22.6%)
Underwent mastectomy	264 (63.0%)	84 (63.2%)
Underwent chemotherapy	342 (81.6%)	114 (85.7%)
Neoadjuvant	113 (27.2%)	42 (31.8%)
Adjuvant	229 (55.2%)	72 (54.6%)
Prescribed hormone therapy	285 (68.0%)	94 (70.7%)
Fertility information		
Pregnancy/births		
Never delivered	103 (24.6%)	65 (48.9%)
Delivered one child	71 (17.0%)	33 (24.8%)
Delivered more than one child	245 (58.5%)	35 (26.3%)
Had children before cancer diagnosis	316 (75.4%)	68 (51.1%)
Number of children before cancer diagnosis: Median [range]	2 [0–5]	1 [0–5]
Never pregnant	85 (20.3%)	54 (40.6%)
History of difficulty becoming pregnant	76 (18.1%)	35 (26.3%)
History of infertility treatments before diagnosis	42 (10.0%)	15 (11.3%)
Not menstruating at time of survey	157 (37.5%)	33 (24.8%)
Menstruating less than once every 12 months	37 (8.8%)	4 (3.0%)

Abbreviations: ER, estrogen receptor; Her2, human epidermal growth factor receptor 2; PR, progesterone receptor; PSS, Perceived Stress Scale.

Among women who indicated any fertility concerns (*n* = 133), the median age was 35 (range, 22–45). Other demographics, tumor/treatment characteristics, and psychosocial measures were comparable to the full study sample. Half of the concerned group had one or more children already at the time of the cancer diagnosis (51%), and 26% had experienced some difficulty becoming pregnant previously.

### 3.1 Fertility concerns, decision‐making, and fertility preservation use

Regarding the changes in fertility concerns over time, the level of concern at 1 year was significantly lower compared to the level of concern at baseline (32%, baseline vs. 23%, 1 year, *p* < 0.001 by McNemar's test).

Table 2 presents fertility concerns, decision‐making, and strategies for preservation and communication among the whole cohort and those defined as concerned. In the population of patients with concerns, a substantial proportion were concerned that a pregnancy would increase the risk of recurrence, whether they wished to have a child or not (55% and 42%, respectively). Other concerns about having children were caring for them if cancer recurred (37% if they wished to have a child, and 21% if not) and the child having an increased risk of developing cancer (45% if they wished to have a child, and 11% if not).

**TABLE 2:** Fertility concerns, decision‐making, and strategies.

Concern	No. of patients (%)	
	Total cohort (*N* = 419)	Concerned cohort (*N* = 133)
Before breast cancer diagnosis, wished to have biologic children in the future	101 (24.1%)	94 (70.7%)
At time of survey, wished to have biologic children in the future	71 (17.0%)	67 (50.4%)
If wanted more children, concerned about^a^	*N* = 71	*N* = 67
Caring for them if cancer recurred	25 (35.2%)	25 (37.3%)
Children having increased risk of developing cancer	31 (43.7%)	30 (44.8%)
Pregnancy would increase risk of recurrence	37 (52.1%)	37 (55.2%)
Other reasons	15 (21.1%)	13 (19.4%)
No answer	15 (21.1%)	13 (19.4%)
If did not want more children, concerned about^a^	*N* = 348	*N* = 66
Decision to have no more biologic children before diagnosis	248 (71.3%)	14 (21.2%)
Caring for them if cancer recurred	24 (6.9%)	19 (28.8%)
Children having increased risk of developing cancer	10 (2.9%)	7 (10.6%)
Pregnancy would increase risk of recurrence	36 (10.3%)	28 (42.4%)
Other reasons	68 (19.5%)	20 (30.3%)
No answer	3 (0.8%)	0 (0%)
Felt pressured by partner, family or friends to have children, somewhat or a lot	19 (4.6%)	18 (13.5%)
Concerns about fertility affected treatment decisions		
Not at all	350 (83.5%)	70 (52.6%)
A little	25 (6.0%)	22 (16.5%)
Somewhat	21 (5.0%)	21 (15.8%)
Very	21 (5.0%)	19 (14.3%)
Missing	2 (0.5%)	1 (0.8%)
How concerns about fertility affected treatment decisions (at 3,6, and 12 months, some patients indicated >1)		
Fertility concerns led patient to choose not to receive chemotherapy	0 (0%)	0 (0%)
Fertility concerns led patient to choose another chemotherapy over another	15 (3.6%)	7 (5.3%)
Fertility concerns led patient to choose not to receive endocrine therapy	8 (1.9%)	6 (4.5%)
Fertility concerns led patient to consider receiving endocrine therapy for <5 years	69 (16.5%)	38 (28.6%)
Other reasons	66 (15.8%)	32 (24.1%)
None of the above	295 (70.4%)	74 (55.6%)
Took special steps to lessen chance of infertility (some patients indicated >1)	29 (6.9%)	27 (20.3%)
Embryo cryopreservation	14 (3.3%)	14 (10.5%)
Oocyte cryopreservation	14 (3.3%)	14 (10.5%)
GnRH agonist	8 (1.9%)	8 (6.0%)
Others	5 (1.2%)	3 (2.3%)
Unsure	22 (5.3%)	7 (5.3%)
Discussed fertility issues with physician before starting therapy	251 (59.9%)	117 (88.0%)

^a^ Some patients indicated >1.

Decision‐making about treatments was reported as affected by fertility concerns to some degree in almost half of the patients with concerns (47%) and 14% of those without concerns. Over the year of follow‐up, using data from the 3‐, 6‐, and 12‐month follow‐up surveys, participants reported various impacts on treatment including 29% of those concerned considering taking fewer than 5 years of endocrine therapy. Standard strategies to preserve fertility were primarily pursued by women who were concerned about fertility, though only 20% of these participants did undergo preservation strategies: 14 underwent cryopreservation of embryos, 14 underwent cryopreservation of eggs, and 8 took a GnRH agonist through chemotherapy.

Overall, 60% of participants discussed fertility concerns with a provider at the time of treatment decision‐making, including 88% of those who indicated fertility concerns, and 87% who reported that this was addressed adequately.

In the multivariate model (Table 3), women with a history of difficulty becoming pregnant had a 3‐fold greater odds of having fertility concerns and younger women had higher odds of fertility concerns. Those who had a child before diagnosis had 81% lower odds of fertility concerns compared with women who did not. While being highly educated was associated with greater fertility concerns and menstruating fewer than once every 12 months at diagnosis was associated with less fertility concerns in the univariate models, they were not statistically significant in the multivariate model. Factors not associated with fertility concerns in univariate models, and thus not included in multivariate models, included race, site, income, marital status, having a first‐degree relative with breast or ovarian cancer, tumor biology (stage, ER/PR receptors, and HER2 expression), treatment, psychosocial measures and history of infertility treatments.

**TABLE 3:** Logistic regression evaluating associations between patient/disease characteristics and fertility concerns.

Variable	Univariate models			Final multivariate model		
	OR	95% CI	*p*	OR	95% CI	*p*
Age						
≤30 vs. 36–45	41.181	12.15 to 139.59	**<0.001**	25.869	7.23 to 92.62	**<0.001**
31–35 vs. 36–45	7.969	4.47 to 14.20	**<0.001**	9.645	5.12 to 18.18	**<0.001**
Non‐Hispanic White vs. not	0.899	0.56 to 1.46	0.666			
College educated vs. not	2.841	1.30 to 6.22	**0.009**			
Academic vs. community site	1.483	0.98 to 2.25	**0.065**			
Income ≥$50,000 vs. <$50,000	0.716	0.45 to 1.14	**0.162**			
Married vs. not	0.718	0.44 to 1.18	**0.191**			
First‐degree relative with breast or ovarian cancer vs. none	1.055	0.61 to 1.83	0.848			
ER/PR positive vs. negative	0.912	0.56 to 1.48	0.710			
Her2 positive vs. negative	0.892	0.55 to 1.46	0.649			
Stage I & II vs. III & IV	1.212	0.66 to 2.22	0.532			
Underwent chemotherapy						
Neoadjuvant vs. not at all	1.807	0.94 to 3.48	**0.077**			
Adjuvant vs. not at all	1.401	0.77 to 2.56	**0.271**			
Underwent mastectomy	0.992	0.65 to 1.52	0.970			
Stress						
Moderate vs. low	0.963	0.62 to 1.49	0.866			
High vs. low	1.670	0.75 to 3.71	0.208			
Anxiety	1.190	0.77 to 1.84	0.436			
Depression	0.976	0.45 to 2.12	0.951			
Had children before diagnosis	0.160	0.10 to 0.26	**<0.001**	0.185	0.11 to 0.33	**<0.001**
History of difficulty becoming pregnant	2.16	1.30 to 3.60	**0.003**	3.013	1.63 to 5.56	**<0.001**
History of infertility treatments before diagnosis	1.225	0.63 to 2.39	0.551			
Menstruating less than once every 12 months before diagnosis	0.234	0.08 to 0.68	**0.007**			

Abbreviations: CI, confidence interval; ER, estrogen receptor; Her2, human epidermal growth factor receptor 2; OR, odds ratio; PR, progesterone receptor.

### 3.2 Factors associated with having a treatment decision affected by fertility concerns and utilization of fertility preservation among patients with fertility concerns

In an exploratory analysis, we examined a number of factors among only those who had fertility concerns at baseline (*n* = 133). Younger age (≤30 vs. 36–45, OR = 5.03, 95% CI: 1.81–14.03; 31–35 vs. 36–45, OR = 3.97, 95% CI: 1.66–9.50) was associated with having a treatment decision affected by fertility concerns, whereas having children was associated with a lower likelihood of having a treatment decision affected (OR = 0.33, 95% CI: 0.15–0.74) in the final multivariate model (Table 4). Other demographic, treatment, and psychosocial characteristics were not significant in either univariate or multivariate models. Women who had already had a child were less likely to undergo any fertility preservation treatment (OR = 0.05, 95% CI: 0.01–0.22). Women age ≤ 30 years (vs. age ≥ 36) were less likely to undergo fertility preservation (OR = 4.71, 95% CI: 1.59–13.99) only in the univariate model. Other demographic, treatment, and psychosocial characteristics were not significant in either univariate or multivariate models (Table 5).

**TABLE 4:** Logistic regression evaluating associations between patient/disease characteristics and treatment decisions being affected by fertility concerns in the concerned cohort (*n* = 133).

Variable	Univariate models			Final multivariate model		
	OR	95% CI	*p*	OR	95% CI	*p*
Age						
≤30 vs. 36–45	6.794	2.53 to 18.25	**0.005**	5.034	1.81 to 14.03	**0.002**
31–35 vs. 36–45	3.451	1.51 to 7.90	0.484	3.970	1.66 to 9.50	**0.002**
Non‐Hispanic White vs. not	0.722	0.33 to 1.60	0.424			
College educated vs. not	2.306	0.43 to 12.33	0.329			
Academic site vs. community site	1.508	0.76 to 3.00	0.242			
Income ≥$50,000 vs. <$50,000	0.889	0.42 to 1.89	0.760			
Married vs. not	1.100	0.49 to 2.47	0.818			
Stress						
Moderate vs. low	0.877	0.42 to 1.82	0.725			
High vs. low	0.893	0.26 to 3.04	0.856			
Anxiety	1.205	0.59 to 2.47	0.610			
Depression	1.768	0.48 to 6.58	0.396			
Had children before diagnosis	0.306	0.15 to 0.62	**0.001**	0.334	0.15 to 0.74	**0.007**
Discussed fertility issues with physician before starting treatment	2.703	0.81 to 8.98	0.104			

Abbreviations: CI, confidence interval; OR, odds ratio.

**TABLE 5:** Logistic regression evaluating associations between patient/disease characteristics and any fertility preservation strategy in the concerned cohort (*n* = 133).

Variable	Univariate models		
	OR	95% CI	*p*
Age			
≤30 vs. 36–45	4.714	1.59 to 13.99	**0.011**
31–35 vs. 36–45	2.042	0.70 to 5.99	0.895
Non‐Hispanic White vs. not	0.585	0.23 to 1.47	0.254
College educated vs. not	–	–	–
From academic site vs. community site	1.122	0.48 to 2.62	0.790
Income ≥$50,000 vs. <$50,000	1.108	0.44 to 2.82	0.830
Married vs. not	0.554	0.22 to 1.40	0.211
ER/PR positive	0.721	0.28 to 1.85	0.498
Her2 positive	0.531	0.17 to 1.69	0.283
Stage I & II vs. III & IV	0.804	0.24 to 2.70	0.723
Underwent chemotherapy			
Neo adjuvant vs. not at all	0.676	0.14 to 3.19	0.621
Adjuvant vs. not at all	1.667	0.43 to 6.43	0.458
Stress			
Moderate vs. low	0.774	0.31 to 1.91	0.578
High vs. low	1.036	0.24 to 4.44	0.962
Anxiety	1.395	0.59 to 3.32	0.453
Depression	1.768	0.43 to 7.34	0.433
Had children before diagnosis	0.048	0.01 to 0.22	**<0.001**
Discussed fertility issues with physician before starting treatment	–	–	–

Abbreviations: CI, confidence interval; ER, estrogen receptor; Her2, human epidermal growth factor receptor 2; OR, odds ratio; PR, progesterone receptor.

## 4 DISCUSSION

Our findings confirm and expand upon the previous literature regarding fertility concerns and their consequences among young women with breast cancer.^11^, ^14^, ^15^, ^18^, ^24^, ^25^ The fact that 32% of the patients had some fertility concerns at the time of treatment decision‐making is somewhat lower than noted in prior studies. In a cross‐sectional analysis of 657 breast cancer survivors (average age at diagnosis was 33 years), 73% of respondents reported at least some degree of fertility concern.^18^ In an analysis of 724 women enrolled in a prospective cohort study, 51% of women (median age of 37 years) reported at least some degree of concerns.^14^ In a prospective study conducted in Europe (*n* = 297), 32% of whom were under 35 years of age, and 64% had at least some fertility concerns.^15^ These collective findings suggest that the lower prevalence observed in our study may in part be attributable to the older age of our population (45% of the participants were aged between 41 and 45 years), with these women more likely to have finished their desired childbearing at the time of diagnosis. It is also possible that this difference may be attributable to the large proportion of patients enrolled from community sites in our study; patients seen at academic sites may have more concerns.

Our finding that experiencing difficulty becoming pregnant in the past and not having had a child were associated with greater concerns about fertility was not unexpected. However, the fact that the youngest participants had the highest odds of concerns might suggest that there is need for additional patient education regarding fertility‐related risks. Current evidence suggests that about 88% of the youngest women will not experience any treatment‐related amenorrhea,^26^ and that only 5% of those diagnosed at age 30 are likely to be infertile after resumption of menses.^27^ Furthermore, our finding in the concerned population that about half were concerned that a pregnancy would increase risk of recurrence, underscores the importance of ensuring patients understand these risks. It should be communicated to patients who are interested in future childbearing that the currently available collective evidence indicates that pregnancy is safe in breast cancer survivors, including those with ER‐positive disease.^28^, ^29^

The high percentage of women reporting that their providers discussed fertility implications with them is consistent with recent literature that demonstrates improved attention to fertility concerns.^14^, ^30^, ^31^, ^32^ Providers generally appear to be following guidelines recommending that fertility be routinely addressed with young cancer patients. Oncofertility counseling to inform patients about the risk of infertility and strategies for preservation of ovarian function and/or fertility is increasingly considered standard of care at the time of diagnosis in all young women with breast cancer. It is notable and reassuring that none of the psychosocial factors was associated with fertility concerns, which is consistent with prior research.^14^

In a previous study by Partridge et al.,^18^ nearly 30% of women interested in future fertility reported consideration of taking hormonal medication for fewer than 5 years. However, in a prospective study of 384 women with stage I to III breast cancer diagnosed at age 40 years and younger, there was no significant difference at 30 months regarding the proportion of “more concerned about fertility” patients between adherers and non‐adherers of hormonal therapy (39% of the adherers were more concerned versus 43% for the non‐adherers, *p* = 0.44).^33^ From this same prospective cohort, Sella et al.^34^ reported that concern about fertility was a contributor to adjuvant endocrine therapy decisions among a substantial proportion of young breast cancer survivors. It is also possible that feelings about fertility and potential impact on treatment decisions may change over time, which we were unable to observe in our study given that we only followed patients out 12 months. Nevertheless, recent data from a prospective clinical trial of temporary interruption of endocrine therapy for pregnancy (the POSITIVE Trial) demonstrated short‐term safety from this approach, which should help to address the concerns and inform the decisions regarding endocrine therapy.^29^

It is recommended that providers be prepared to discuss fertility preservation options and/or to refer all potential patients to appropriate reproductive specialists,^6^ yet only one‐fifth of those concerned about fertility underwent fertility preservation. This low rate is consistent with prior reports including that of Lambertini et al.^35^ that demonstrated that only a minority of young breast cancer patients (≤40 years of age) accessed a fertility unit to discuss preservation procedures (29%), and even fewer (12%) ultimately decided to undergo one of the available cryopreservation strategies. This was comparable with the data from an earlier prospective cohort study^14^: Despite the fact that half of the cohort presented some fertility concerns, only 8.5% accessed one of the available cryopreservation procedures. Race, income, education, community versus academic site, and undergoing neoadjuvant chemotherapy did not predict that utilization of fertility preservation strategies. It is important to highlight that these data were collected back in 2012–2013. Since then, access and options to fertility preservation have evolved favorably in the U.S., including the emergence of state‐based fertility preservation insurance mandates.^36^ However, a significant number of barriers to accessing oncofertility care remain, including lack of financial or geographic access,^37^ lack of information and referral, and personal fear or uncertainty regarding fertility presentation. While we were unable to evaluate specific reasons for non‐utilization in our analysis, we believe that medical insurance coverage and cost of the procedures were factors that may have discouraged patients from pursuing these procedures, as demonstrated in prior studies.^38^ All of the patients in this study had insurance, and we did not assess other barriers to the use of fertility preservation.

The present study has several important limitations. While the trial was conducted at sites across the U.S. and clinics were asked to recruit all eligible patients systematically at their first visit, patients who enrolled may not be fully representative of all patients with premenopausal breast cancer. For example, the proportion with college education was very high (88%), limiting generalizability. In addition, the relatively small sample of “concerned” patients may hinder the detection of weak associations. However, strengths of the present study include the enrollment and baseline assessment of fertility concerns within 3 months following diagnosis, minimizing the recall bias that impacted prior studies of this issue among longer‐term survivors. Moreover, our population included patients from 14 academic and 40 community practices geographically distributed across the U.S. Further, the 90% survey response rate was quite high for our main outcome.

## 5 CONCLUSION

This large, prospective, geographically, and racially/ethnically diverse study confirms and expands upon existing knowledge regarding fertility concerns and their consequences for treatment decision‐making and fertility preservation strategies among young women with breast cancer. As a significant proportion of women are concerned about fertility at diagnosis that may impact their treatment decisions, it is critical to ensure that these issues are adequately addressed.

## AUTHOR CONTRIBUTIONS

**Elisabeth de Kermadec:** Conceptualization (equal); data curation (equal); investigation (equal); methodology (equal); visualization (equal); writing – original draft (equal); writing – review and editing (equal). **Yue Zheng:** Data curation (equal); formal analysis (lead); methodology (equal); software (lead); validation (lead); visualization (equal); writing – original draft (equal); writing – review and editing (equal). **Shoshana Rosenberg:** Methodology (equal); writing – original draft (equal); writing – review and editing (equal). **Kathryn J. Ruddy:** Writing – review and editing (equal). **Jennifer A. Ligibel:** Writing – review and editing (equal). **Karen M. Emmons:** Funding acquisition (equal); methodology (equal); writing – review and editing (equal). **Ann H. Partridge:** Conceptualization (equal); data curation (equal); funding acquisition (equal); investigation (equal); methodology (equal); project administration (lead); resources (lead); supervision (lead); writing – review and editing (equal).

## FUNDING INFORMATION

This work was supported by an American Society of Clinical Oncology Conquer Cancer Foundation/Susan G. Komen Improving Cancer Care grant (Principal Investigator: Ann H. Partridge), a Susan G. Komen grant (Principal Investigator: Ann H. Partridge), a Breast Cancer Research Foundation grant (Principal Investigator: Ann H. Partridge), and by the National Institutes of Health grant 5K05 CA124415‐05 (Principal Investigator: Karen M. Emmons). Study‐related materials were developed in partnership with the Dana‐Farber/Harvard Cancer Center Health Communication Core.

## CONFLICT OF INTEREST STATEMENT

AP and KJR receive royalties for co‐authoring the Breast Cancer Survivorship section of UpToDate. All other authors have no conflicts to report.

## Data Availability

The datasets generated during and/or analyzed during the current study are available from the corresponding author on reasonable request.
